# Long Noncoding RNA MALAT1 Promotes Colorectal Cancer Progression by Acting as a ceRNA of miR-508-5p to Regulate RAB14 Expression

**DOI:** 10.1155/2020/4157606

**Published:** 2020-12-04

**Authors:** Chen Zhang, Kunhou Yao, Junjie Zhang, Chenyu Wang, Chaoyang Wang, Changjiang Qin

**Affiliations:** ^1^First Department of Oncology, Xinxiang Central Hospital, Xinxiang, 453000 Henan, China; ^2^Department of General Surgery, Huaihe Hospital of Henan University, Kaifeng, 475000 Henan, China

## Abstract

Accumulating evidence suggested that lncRNA MALAT1 plays critical roles in the commencement and progression of malignant cancers. Nevertheless, the function of MALAT1 in colorectal cancer (CRC) remains largely unknown. In the present study, we reported that MALAT1 expression is significantly upregulated in CRC and correlated with advanced TNM stage, lymph node metastasis, and worse prognosis in patients. Functional assays revealed that MALAT1 knockdown reduced CRC cell growth and invasion abilities *in vitro*. Mechanistically, we discovered that MALAT1 may serve as a competing endogenous RNA (ceRNA) to miR-508-5p in CRC progression. Bioinformatics analysis and luciferase assays confirmed that RAB14 acts as a target of miR-508-5p. In addition, downregulation of RAB14 reduced the progression of CRC. Collectively, our findings indicated that MALAT1 could promote CRC progress by sponging miR-508-5p and enhancing RAB14 expression, which provides a therapeutic target in CRC treatment.

## 1. Introduction

Colorectal cancer (CRC) is one of the most prevalent malignancies worldwide [1, 2]. Although the prognosis has ameliorated due to advances in both diagnosis and treatment of CRC, the molecular mechanisms that lead to the development of the tumor remain unclear [3, 4]. Therefore, identification of molecular mechanisms underlying CRC progression is of utmost importance in order to find novel therapy targets for CRC patients.

Long noncoding RNAs (lncRNAs) are a type of ncRNA with more than 200 nucleotides (nt) with limited protein-coding ability [5]. Recently, accumulating data indicated that aberrant lncRNA expression has important functions in diverse biological processes, including growth, differentiation, autophagy, and metastasis [6, 7]. For example, Zhu et al. [8] demonstrated that lncRNA ANCR decreased osteoblast differentiation by regulating the EZH2/Runx2 axis. Xiong et al. [9] revealed that lncRNA HULC triggered autophagy and chemosensitivity in liver cancer by regulating the expression of Sirt1. Sun et al. [10] demonstrated that lncRNA HOXA11-AS encouraged gastric cancer cell growth and invasion by regulating DNMT1, LSD1, and PRC2 expression.

Metastasis-associated lung adenocarcinoma transcript 1 (MALAT1) is first demonstrated by Ji and colleagues as an oncogene in non-small-cell lung cancer through the promotion of cell metastasis and invasion [11]. Recently, dysregulation of MALAT1 was discovered among many other cancers. For example, Zhang et al. [12] indicated that MALAT1 overexpression is connected with advanced clinical features and a worse overall survival rate in renal cell carcinoma. Wu et al. [13] discovered that MALAT1 encourages gallbladder cancer cell proliferation and invasion by regulating the ERK/MAPK axis. Xie et al. [14] demonstrated that MALAT1 increases bladder cancer cell invasion ability by regulating miR-125b expression. Nevertheless, the mechanism of MALAT1 in CRC advancement is still uncertain.

## 2. Materials and Methods

### 2.1. Tissue Specimens and Ethics Statement

The study was granted approval by the Ethics Committee of Xinxiang Central Hospital and Huaihe Hospital, and all participants granted their informed consent. A total of 43 paired (24 males and 19 females; 51.3 ± 13.81 and 58.7 ± 8.97 years old) tissue samples were gathered from patients who were diagnosed with CRC between Jan 1, 2015, and Dec 1, 2016. No participants were administered radiotherapy or chemotherapy before the operation. All tissue samples were kept at −80°C until use (Table 1).

### 2.2. Cell Culture and Transfection

Human CRC (SW480, HT-29, SW620, LoVo, HCT-8) and normal colon epithelial cell line (FHC) were acquired from American Type Culture Collection (ATCC, Manassas, VA, USA). Cells were grown in Dulbecco's modified Eagle's medium (DMEM; Invitrogen, Carlsbad, CA, USA) accompanied by 10% fetal bovine serum (FBS) and placed at 37°C in 5% CO_2_.

MALAT1 cDNA was cloned into pcDNA3.1 (Invitrogen) to construct a vector that would lead to overexpression of MALAT1 (pcDNA3.1/MALAT1) according to a previous study [15]. The miR-508-5p mimics, MALAT1 siRNA (si-MALAT1-1 sequence is 5′-CACAGGGAAAGCGAGTGGTTGGTAA-3′, si-MALAT1-2 sequence is 5′-GAGGUGUAAAGGGAUUUAUTT3′, and si-MALAT1-3 sequence is 5′-CACAGGGAAAGCGAGUGGUUGGU-3′), RAB14 siRNA, and scramble negative controls were ordered from GenePharma (Shanghai, China). Cells were seeded in 6-well plates (5 × 10^4^ cells/well) and incubated for 24 h before being transfected with the above-mentioned oligonucleotides and plasmids (30 nM) using Lipofectamine 2000 (Invitrogen, Carlsbad, CA, USA) according to the manufacturer's instructions.

### 2.3. Quantitative Real-Time RT-PCR

Total RNA was isolated using a TRIzol reagent (Invitrogen). RNA was reverse transcribed to cDNA by utilizing a reverse transcription kit (TaKaRa, China). The PrimeScript™ RT Reagent Kit (TaKaRa, China) was utilized to identify mRNA expression. U6 or GAPDH served as an endogenous control. The relative fold expression was measured by utilizing the 2^-*ΔΔ*Ct^ method. Primer sequences were as follows: MALAT1, 5′-AAAGCAAGGTCTCCCCACAA-3′ (forward) and 5′-GGTCTGTGCTAGATCAAAAGGCA-3′ (reverse); miR-508-5p, 5′-ACACTCCAGCTGGGTACTCCAGAGGGCGTCACT-3′ (forward) and 5′-TGGTGTCGTGGAGTCG-3′ (reverse); and RAB14, 5′-GCAGATTTGGGATACAGCAGGG-3′ (forward) and 5′-CAGTGTTTGGATTGGTGAGATTCC-3′.

### 2.4. Western Blot

The procedure was carried out as previously described (13). Briefly, cells or tissues were lysed, and protein concentrations were measured using the BCA Protein Assay Kit (Pierce, Rockford, IL, USA). Protein samples were resolved by SDS-polyacrylamide gel electrophoresis and transferred to nitrocellulose membranes. The membranes were incubated with anti-RAB14 (1 : 1000, ab28639, Abcam, CA, USA) and anti-GAPDH (1 : 5000, ab181602, Abcam, CA, USA). After washing, the membranes were then incubated with secondary antibodies coupled to horseradish peroxidase for 1 h at room temperature. Protein signals were detected by the Western Lightning Plus ECL kit (PerkinElmer, Waltham, MA, USA) and quantified by densitometry.

### 2.5. Luciferase Reporter Assay

Wild-type or mutant MALAT1 (MALAT1-WT or MALAT1-MUT) and 3′UTR of RAB14 ligated to pGL3-basic vector (Promega, Madison, WI, USA) were used to build pGL3-MALAT1-WT, pGL3-MALAT1-MUT, pGL3-RAB14-WT, and pGL3-RAB14-MUT. Then, these plasmid vectors were cotransfected with miR-508-5p mimics into HEK293T cells. After 48 h of transfection, cells were harvested for the luciferase reporter assay with a Dual-Luciferase Reporter Assay System (Promega). Renilla luciferase was used as an internal reference.

### 2.6. CCK-8 Assay

Cell Counting Kit-8 (CCK-8, Dojindo, Japan) was used to detect cell proliferation. Briefly, transfected cells were added onto 96-well plates (5 × 10^3^ cells/well). Then, 10 *μ*l CCK-8 reagents were placed into every well at 24, 48, and 72 h, respectively. Finally, the optical density (OD) was quantified at 450 nm through a microplate reader (BioTek, VT, USA).

### 2.7. Colony Formation Assay

Six-well plates with transfected cells (2000 cells/well) were maintained for 2 weeks. Then, the colonies were fixed with 4% paraformaldehyde for 30 min and stained with 0.1% crystal violet for 10 min. The clones were imaged by using a microscope (Nikon, Japan).

### 2.8. Transwell Invasion Assay

An invasion assay was carried out by utilizing transwell chamber inserts (24 wells, 8 *μ*m pore size, Corning, NY, USA) which were precoated with Matrigel (BD Biosciences, San Jose, CA, USA), and transfected cells (2 × 10^4^ cells/well) were added to the upper chamber. Added to the lower chamber was 500 *μ*l DMEM containing 15% FBS. Cells in the upper chamber were removed after incubation for 24 h, and cells that invaded the lower membrane surface were fixed with 4% paraformaldehyde and stained with 0.1% crystal violet solution [12].

### 2.9. RNA Immunoprecipitation (RIP) Assay

According to the manufacturer's protocol, the Magna RIP™ RNA-Binding Protein Immunoprecipitation Kit (Millipore, MA, USA) was applied to conduct the RIP assay by using Ago2 antibody (Abcam) or IgG antibody (negative control). And the immune-precipitated RNAs were finally confirmed using qRT-PCR.

### 2.10. Statistical Analysis

Results from a minimum of 3 independent experiments were statistically analyzed by utilizing SPSS 19.0. Data was expressed as the mean ± standard deviation (SD). The data were analyzed either by two-tailed unpaired Student's *t*-test (two groups) or by one-way ANOVA (greater than two groups). *p* values ≤ 0.05 were regarded as statistically significant.

## 3. Results

### 3.1. lncRNA MALAT1 Is Highly Expressed in CRC

**Figure 1:**
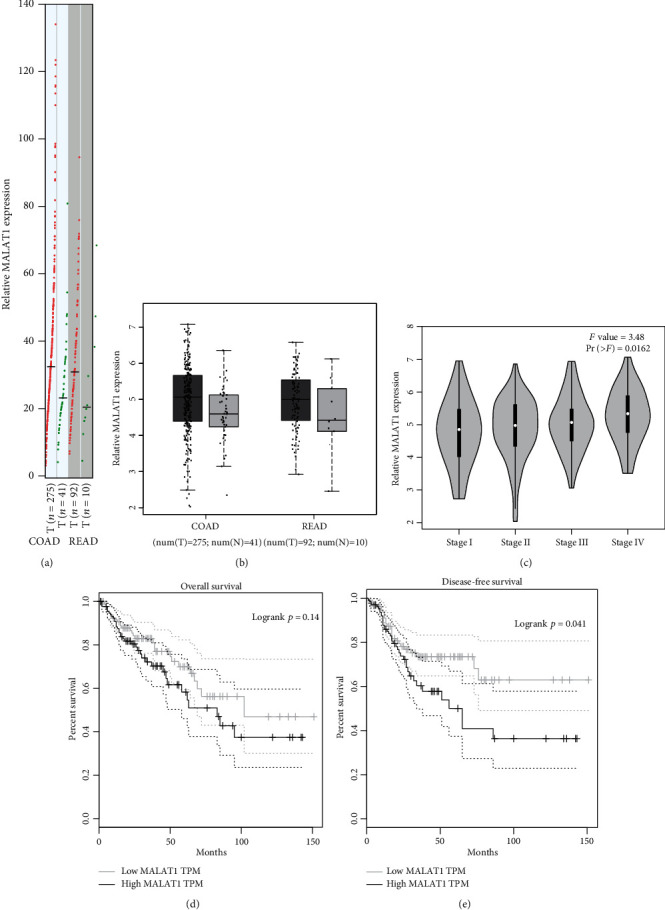
MALAT1 is dysregulated in CRC. (a, b) GEPIA database demonstrated that MALAT1 levels are upregulated in CRC tissues. (c) High MALAT1 levels were correlated with CRC tumor stage. (d) High MALAT1 was correlated with low overall survival in CRC. (e) High MALAT1 was correlated with poor disease-free survival in CRC. COAD: colon adenocarcinoma; READ: rectum adenocarcinoma; T: tumor tissues; N: normal tissues; ^∗^*p* < 0.05.

In order to discover the function of MALAT1 in CRC tumorigenesis, analysis of the GEPIA database was performed. Data indicated that MALAT1 was significantly upregulated in colon adenocarcinoma (COAD) in comparison with normal samples. Similarly, MALAT1 was also higher in rectum adenocarcinoma (READ) (Figures 1(a) and 1(b)). High MALAT1 was significantly correlated with an advanced tumor stage in CRC patients (Figure 1(c)). GEPIA analysis showed that patients with high MALAT1 levels were associated with a poor overall survival (OS) rate (Figure 1(d)). Moreover, high MALAT1 levels were significantly associated with the disease-free survival (DFS) rate in CRC patients (Figure 1(e)).

**Figure 2:**
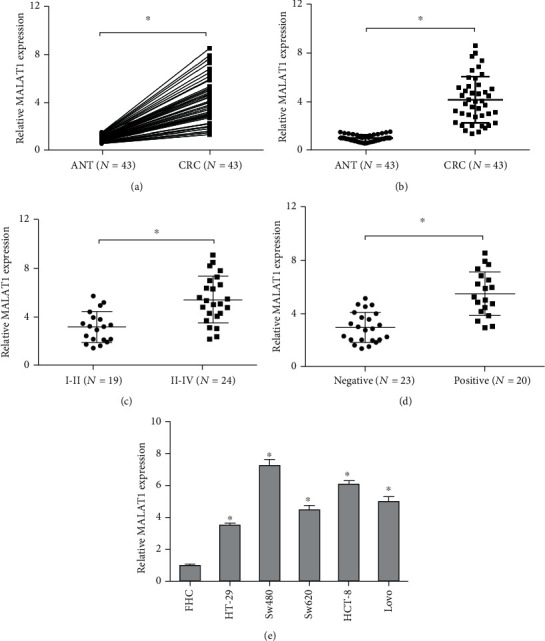
MALAT1 is highly expressed in CRC. (a, b) The MALAT1 levels in CRC tissues were evaluated using qRT-PCR. (c, d) High MALAT1 levels were correlated with advanced TNM stage and lymph node metastasis in CRC patients. (e) MALAT1 expression was upregulated in CRC cells compared to normal colon epithelial cell line (FHC). ANT: adjacent nontumor tissues; CRC: colorectal cancer. ^∗^*p* < 0.05.

We further investigated MALAT1 levels in CRC tissues. qRT-PCR results demonstrated that MALAT1 expression was substantially increased in CRC tissues (Figures 2(a) and 2(b)). High MALAT1 levels were associated with an advanced TNM stage and lymph node metastasis in CRC patients (Figures 2(c) and 2(d)). In addition, MALAT1 expression was significantly upregulated in CRC cell lines (SW480, HT-29, SW620, LoVo, and HCT-8) compared to the normal colon epithelial cell line (FHC) (Figure 2(e)).

### 3.2. Knockdown of MALAT1 Suppressed CRC Cell Proliferation and Invasion

**Figure 3:**
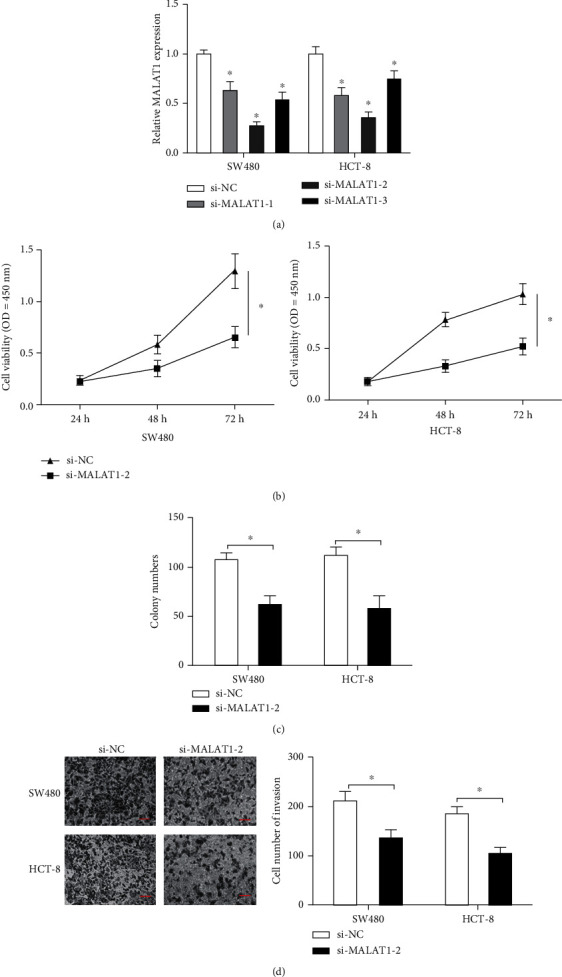
Knockdown of MALAT1 decreases the proliferation and invasion ability of CRC in vitro. (a) MALAT1 expression in SW480 and HCT-8 cells transfected with si-MALAT1 or si-NC. (b, c) MALAT1 inhibition decreased SW480 and HCT-8 cell proliferation and colony formation ability in vitro. (d) MALAT1 knockdown inhibited SW480 and HCT-8 cell invasion ability in vitro. ^∗^*p* < 0.05.

To investigate the biological consequences of MALAT1 in CRC, we suppressed MALAT1 expression in SW480 and HCT-8 cells (Figure 3(a)). CCK-8 and colony formation experiments demonstrated that MALAT1 inhibition substantially decreased SW480 and HCT-8 cell proliferation abilities *in vitro* (Figures 3(b) and 3(c)). The transwell assay reported that MALAT1 knockdown inhibited SW480 and HCT-8 cell invasion abilities *in vitro* (Figure 3(d)).

### 3.3. MALAT1 Binds Directly to miR-508-5p to Facilitate CRC Progression

**Figure 4:**
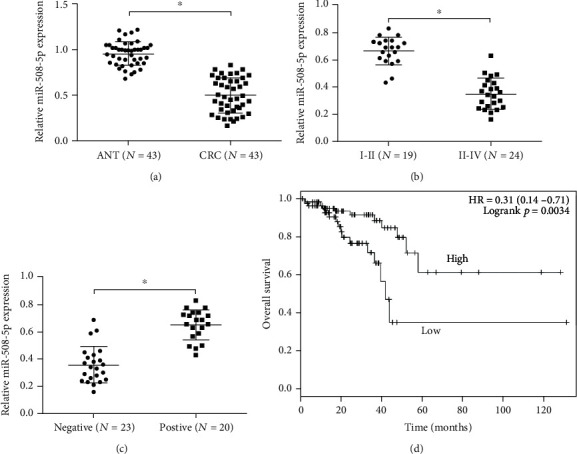
miR-508-5p expression is downregulated in CRC. (a) The expression of miR-508-5p was decreased in CRC tissues. (b, c) Low miR-508-5p expression was inversely correlated with advanced TNM stage and lymph node metastasis. (d) Low miR-508-5p expression was correlated with worse overall survival in CRC patients. ANT: adjacent nontumor tissues; CRC: colorectal cancer; ^∗^*p* < 0.05.

Next, we explored miR-508-5p levels in CRC. qRT-PCR results demonstrated that miR-508-5p levels were significantly reduced in and correlated with advanced TNM stage and lymph node metastasis in CRC patients (Figures 4(a)–4(c)). Kaplan-Meier analysis demonstrated that CRC patients with low miR-508-5p expression had a poor overall survival compared to patients with high miR-508-5p expression (Figure 4(d)).

**Figure 5:**
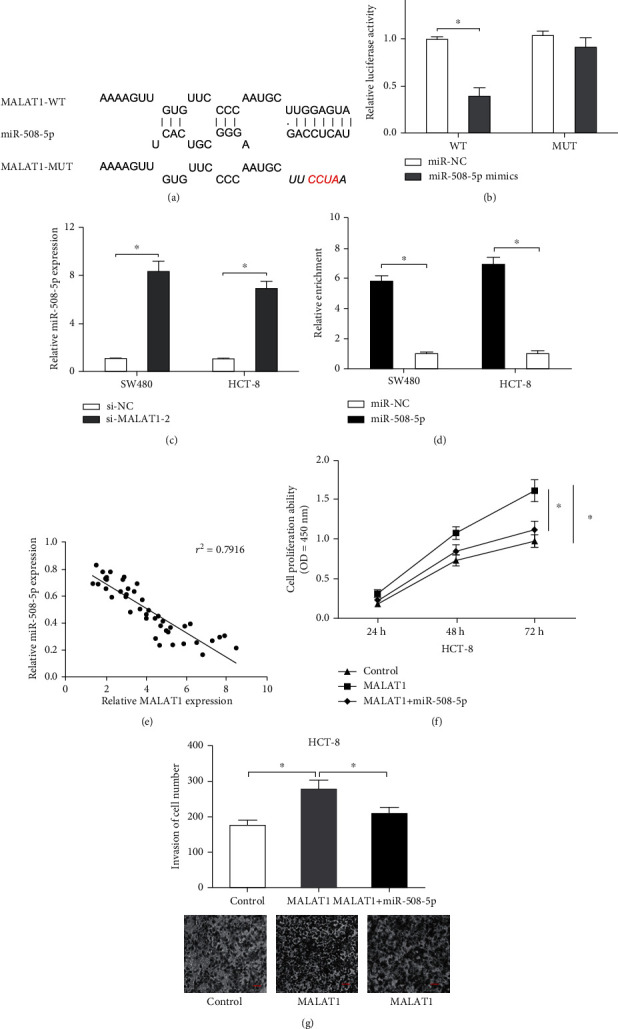
MALAT1 functions as the sponge of miR-508-5p in CRC cells. (a) Predicted binding sites in MALAT1 for miR-508-5p. (b) miR-508-5p mimics decreased the luciferase activity of the MALAT1-WT group. (c) MALAT1 inhibition increased miR-508-5p expression in SW480 and HCT-8 cells. (d) RIP assay revealed that the enrichment of MALAT1 was highly elevated by miR-508-5p overexpression in SW480 and HCT-8 cells. (e) MALAT1 expression was inversely associated with miR-508-5p expression in CRC tissues. (f, g) miR-508-5p mimics rescued the effects of MALAT1 overexpression on HCT-8 cell proliferation and invasion. ^∗^*p* < 0.05.

Recent evidence shows that certain lncRNAs sponge the biological functions of miRNAs by acting as competing endogenous RNAs (ceRNAs) [15]. DIANA online software suggested the existence of miR-508-5p binding sites in MALAT1 (Figure 5(a)). The luciferase reporter assay identified that miR-508-5p mimics significantly decreased the luciferase activity of the MALAT1-WT vector (Figure 5(b)). qRT-PCR showed that si-MALAT1 increased miR-508-5p expression in SW480 and HCT-8 cells (Figure 5(c)). The RIP assay further confirmed the correlation between MALAT1 and miR-508-5p in CRC (Figure 5(d)). Spearman's correlation analysis indicated that MALAT1 expression was inversely correlated with miR-508-5p in CRC tissues (Figure 5(e)). Furthermore, rescue assays indicated that MALAT1 overexpression on HCT-8 cell proliferation and invasion could be hindered by miR-508-5p mimics (Figures 5(f) and 5(g)).

### 3.4. MALAT1 Encourages CRC Progression by Targeting the miR-508-5p/RAB14 Axis

**Figure 6:**
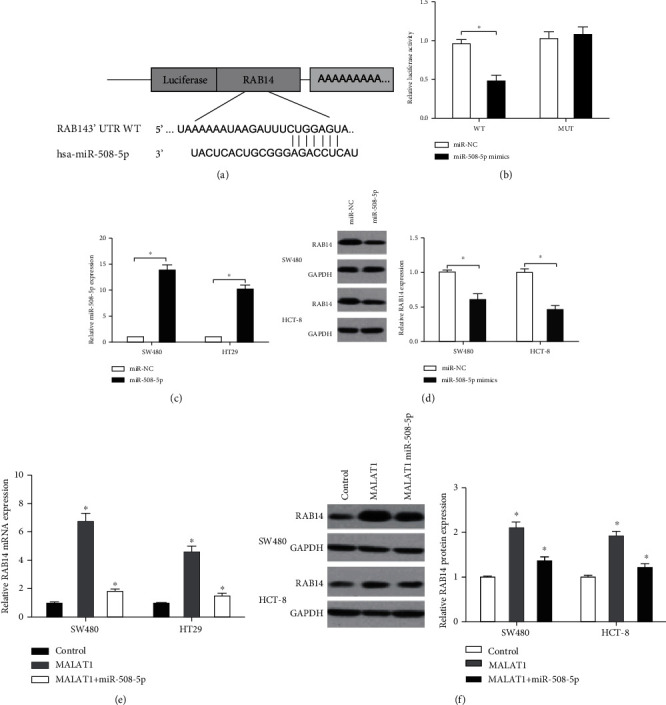
MALAT1 knockdown suppresses RAB14 expression by interacting with miR-508-5p. (a) Predicted binding sites in 3′UTR-RAB14 for miR-508-5p. (b) miR-508-5p mimics decreased the luciferase activity of the WT-RAB14 group. (c) miR-508-5p mimics increased miR-508-5p levels in SW480 and HCT-8 cells. (d) miR-508-5p overexpression reduced RAB14 protein expression in SW480 and HCT-8 cells. (e, f) miR-508-5p mimics abolished the effects of MALAT1 overexpression on RAB14 expression in SW480 and HCT-8 cells. ^∗^*p* < 0.05.

Previous studies indicate that miRNAs can bind the 3′UTR of target genes to control their expression [16]. In the present study, we identified RAB14 as a target of miR-508-5p by TargetScan (Figure 6(a)). The luciferase reporter assay indicated that miR-508-5p mimics decreased the luciferase activity of the RAB14-WT group (Figure 6(b)). Western blot indicated that miR-508-5p mimics decreased RAB14 levels in CRC cells (Figures 6(c) and 6(d)). Furthermore, we showed that MALAT1 overexpression increased RAB14 expression in CRC cells, while miR-508-5p mimics abolished the effects (Figures 6(e) and 6(f)).

**Figure 7:**
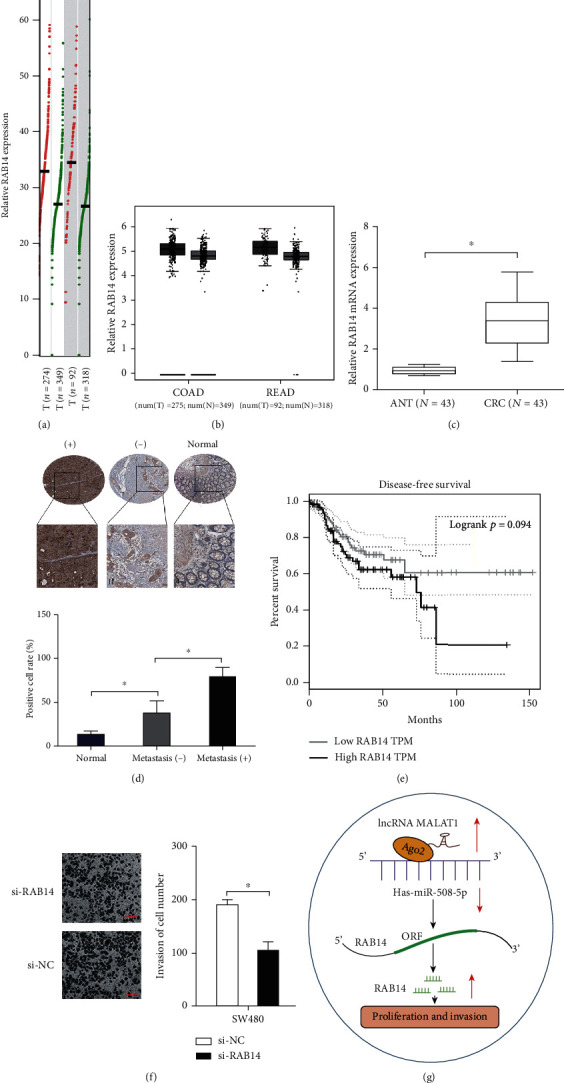
RAB14 is highly expressed in CRC. (a, b) GEPAI analysis determined RAB14 expression in CRC tissues. (c, d) RAB14 expression in CRC tissues was determined by qRT-PCR and IHC analysis. (e) High RAB14 levels were correlated with poor disease-free survival in CRC patients. (f) RAB14 knockdown reduced SW480 cell invasion ability. (g) MALAT1 promoted CRC progression by targeting the miR-508-5p/RAB14 axis. COAD: colon adenocarcinoma; READ: rectum adenocarcinoma; ANT: adjacent nontumor tissues; CRC: colorectal cancer; T: tumor tissues; N: normal tissues; ^∗^*p* < 0.05.

Next, we investigated the roles of RAB14 in CRC progression. GEPIA analysis [17] demonstrated that RAB14 levels were significantly upregulated in CRC (Figures 7(a) and 7(b)). And the results were further validated in 43 pairs of CRC tissues (Figure 7(c)). IHC demonstrated that RAB14 was considerably higher in CRC and associated with metastasis (Figure 7(d)). Kaplan-Meier analysis showed that high RAB14 levels were correlated with a poor disease-free survival rate in CRC patients (Figure 7(e)). Furthermore, the transwell assay indicated that RAB14 inhibition substantially reduced SW480 cell invasion ability *in vitro* (Figure 7(f)). Thus, these data indicated that MALAT1 could promote CRC progression by regulating the miR-508-5p/RAB14 axis (Figure 7(g)).

## 4. Discussion

Accumulating data shows that lncRNAs play critical roles in CRC development [18]. For example, Liu et al. [19] identified that lncRNA DANCR expression is highly expressed in CRC and correlated with advanced clinical features and worse overall survival. Wang et al. [20] suggested that lncRNA AB073614 regulates CRC cell growth and metastasis via the PI3K/AKT axis. Han et al. [21] reported that lncRNA CRNDE encourages chemoresistance and proliferation by the miR-181a-5p/Wnt/*β*-catenin axis in CRC.

Recently, a number of studies have shown that MALAT1 might be a biomarker in CRC progression. For example, Yang et al. [22] discovered that MALAT1 promotes CRC growth and invasion by regulating AKAP-9. Ji et al. [23] discovered that resveratrol inhibits CRC cell invasion and metastasis through the Wnt/*β*-catenin axis. Zhuang et al. [24] demonstrated that MALAT1 encourages CRC cell metastasis by the miR-106b-5p/SLAIN2 axis. In this study, we demonstrated that MALAT1 was amplified and correlated with an advanced TNM stage, lymph node metastasis, and worse prognosis in CRC patients. MALAT1 inhibition reduced CRC cell growth and invasion abilities *in vitro*. Thus, we suggested that MALAT1 may encourage the progression of CRC malignancy, which is consistent with previous studies.

Recent studies showed that certain lncRNAs exert their functions partly by acting as ceRNAs and modulating the expression of miRNA targets. In this study, bioinformatics tools indicated that miR-508-5p serves as a target of MALAT1, which was additionally validated using luciferase reporter and RIP assays. Subsequently, rescue assays demonstrated that miR-508-5p mimics abolished the influence of MALAT1 overexpression on CRC progression. Previous studies showed that miR-508-5p acts as a tumor suppressor in many tumors. For example, Wu et al. [25] discovered that miR-508-5p reduces hepatocellular carcinoma cell progression via regulating MESDC1 levels. Bao et al. [26] demonstrated that miR-508-5p suppresses glioma cell growth by targeting GPNMB. Shang et al. [27] proposed that the miR-508-5p/ABCB1 axis regulates gastric cancer multidrug resistance. Therefore, we suggest that MALAT1 might exert its oncogenic function in CRC progression at least partially by controlling miR-508-5p levels.

RAB14 is a small GTPase member of the RAS oncogene family, which contains more than 170 members, and is divided into five subfamilies—RAS, RAB, RHO, ARF, and RAN [28]. Recent studies suggest that RAB14 might act as an important regulator in tumor advancement. For example, Guo et al. [29] indicated that RAB14 promotes gastric cancer cell proliferation by controlling the AKT pathway. Wang et al. [30] proposed that miR-451 hindered lung cancer advancement by targeting RAB14. Moreover, Wang et al. [31] found that miR-490-3p hindered CRC progression by targeting RAB14. In our study, we showed that RAB14 was substantially upregulated and correlated with lymph node metastasis and poor prognosis in CRC patients. Subsequently, RAB14 was identified as a target of miR-508-5p. Moreover, RAB14 levels were significantly increased by MALAT1 overexpression, and the upregulation could be abolished by miR-508-5p mimics. Thus, our study suggested that MALAT1 might serve as a ceRNA to promote RAB14-mediated CRC progression by regulating miR-508-5p expression.

In conclusion, this work identified that MALAT1 promotes CRC progression by promoting RAB14 via inhibiting miR-508-5p expression. Thus, MALAT1 might serve as a useful biomarker and therapeutic target in CRC treatment.

## Figures and Tables

**Table 1:** Clinicopathological features of CRC patients.

Patient characteristics	Group	Total
Gender	Male	24
	Female	19
Age (years)	<60	14
	≥60	29
Tumor size (cm)	<5 cm	21
	≥5 cm	22
TNM stage	I/II	19
	III/IV	24
Lymph node metastasis	Negative	23
	Positive	20

## Data Availability

All data used to support the findings of this study are included within the article.
